# Supplementary material for: LeafAI: Interpretable plant disease detection for edge computing
Source: PLoS One. 2026 Jan 23;21(1):e0335956. doi: 10.1371/journal.pone.0335956 (PMC12829967; doi:10.1371/journal.pone.0335956)
Supplement: Attachment — Submitted filename: Response to Reviewers.docx [file pone.0335956.s002.docx]

**Response to Reviewers**

**Journal Requirements:**

1. Please ensure that your manuscript meets PLOS ONE's style requirements, including those for file naming. The PLOS ONE style templates can be found at

<https://journals.plos.org/plosone/s/file?id=wjVg/PLOSOne_formatting_sample_main_body.pdf> and

<https://journals.plos.org/plosone/s/file?id=ba62/PLOSOne_formatting_sample_title_authors_affiliations.pdf>

**Response:** We regret that the template was not matched earlier; however, we have adhered to the styling and format options as mentioned in the provided links.

2. Please note that PLOS ONE has specific guidelines on code sharing for submissions in which author-generated code underpins the findings in the manuscript. In these cases, we expect all author-generated code to be made available without restrictions upon publication of the work. Please review our guidelines at <https://journals.plos.org/plosone/s/materials-and-software-sharing#loc-sharing-code> and ensure that your code is shared in a way that follows best practice and facilitates reproducibilitxy and reuse.

**Response:** We have added the direct link to the code in the methodology section, and we have mentioned the same in our data availability statement as per the policy.

3. We note that the grant information you provided in the ‘Funding Information’ and ‘Financial Disclosure’ sections do not match.

   When you resubmit, please ensure that you provide the correct grant numbers for the awards you received for your study in the ‘Funding Information’ section.

**Response:** We have added the funding information section at the end.

4. Thank you for stating the following financial disclosure:
   “We would like to extend our appreciation to King Saud University for funding this work through the Researcher Supporting Project (RSP2025R481), King Saud University, Riyadh, Saudi Arabia”

Please state what role the funders took in the study. If the funders had no role, please state: "The funders had no role in study design, data collection and analysis, decision to publish, or preparation of the manuscript."
If this statement is not correct you must amend it as needed.
Please include this amended Role of Funder statement in your cover letter; we will change the online submission form on your behalf.

**Response:** The funder has took role in preparing the finalized draft during the first draft. Also during the revision, funder helped in revising the manuscript effectively.

5. Thank you for stating the following in the Acknowledgments Section of your manuscript:
   “We would like to extend our appreciation to King Saud University for funding this work through the Researcher Supporting Project (RSP2025R481), King Saud University, Riyadh, Saudi Arabia”

We note that you have provided funding information that is not currently declared in your Funding Statement. However, funding information should not appear in the Acknowledgments section or other areas of your manuscript. We will only publish funding information present in the Funding Statement section of the online submission form.
Please remove any funding-related text from the manuscript and let us know how you would like to update your Funding Statement. Currently, your Funding Statement reads as follows:
“The author(s) received no specific funding for this work”

Please include your amended statements within your cover letter; we will change the online submission form on your behalf.

**Response:** We have added the funding information section at the end.

6. When completing the data availability statement of the submission form, you indicated that you will make your data available on acceptance. We strongly recommend all authors decide on a data sharing plan before acceptance, as the process can be lengthy and hold up publication timelines. Please note that, though access restrictions are acceptable now, your entire data will need to be made freely accessible if your manuscript is accepted for publication. This policy applies to all data except where public deposition would breach compliance with the protocol approved by your research ethics board. If you are unable to adhere to our open data policy, please kindly revise your statement to explain your reasoning and we will seek the editor's input on an exemption. Please be assured that, once you have provided your new statement, the assessment of your exemption will not hold up the peer review process.

**Response:** We have added the direct link to the data in the methodology section, and we have mentioned the same in our data availability statement as per the policy.

7. We note you have included a table to which you do not refer in the text of your manuscript. Please ensure that you refer to **Table 5** in your text; if accepted, production will need this reference to link the reader to the Table.\

**Response**: Thank you for highlighting this issue, it has been addressed in the revised manuscript.

**Recommendations for Revision**

1. Add a simplified schematic or flowchart of the hybrid model for readers to quickly understand the pipeline.

**Response**: In the methodology section, a general workflow is added. In Hybrid decision model hybrid workflow model diagram is added.

2. Edge computing involves low-powered device like Raspberry Pi. Laptops are more pwerful device to deploy deep learning models. To validate the practical applicability of the proposed model in real-world field conditions, it is strongly recommended to deploy and test the system on a true edge computing device such as a Raspberry Pi 4, NVIDIA Jetson Nano, or Google Coral Edge TPU. These platforms offer limited computational resources and power consumption profiles that more accurately reflect the constraints of agricultural edge environments.

The trained DenseNet model can be optimized through quantization (e.g., using TensorFlow Lite or ONNX format) and deployed on the selected edge hardware. Real-time inference tests should be conducted using a live camera feed, capturing leaf images under natural lighting. Key performance metrics such as inference latency, memory usage, CPU utilization, and energy efficiency should be recorded and compared to the laptop-based benchmarks. This would ensure the system meets the operational requirements for real-time, low-power plant disease detection in the field.

**Response** : Thank you for the valuable suggestion. We fully agree that testing on low-powered edge devices like the Raspberry Pi 4, NVIDIA Jetson Nano, or Google Coral Edge TPU provides essential insight into real-world deployment scenarios. However, the primary aim of our research is to develop a practical and sustainable solution that farmers in low-income regions can adopt, in line with the United Nations Sustainable Development Goals (SDGs).

To that end, we deliberately chose a general-purpose, commodity laptop as our deployment platform. While not as resource-constrained as typical edge boards, it balances accessibility, affordability, and computational capability, making it a feasible choice for real-world use without cloud infrastructure. Since the model runs entirely on the device without server support, we consider it a valid edge deployment scenario.

Additionally, using specialized hardware or deploying clusters to meet strict performance metrics, such as energy efficiency or latency under minimal resources, could increase cost and complexity. This runs counter to our goal of creating a scalable, low-cost solution.

In response to the reviewer’s point, we have revised the discussion section to include key performance metrics—inference latency, memory usage, and CPU utilization—measured on the selected laptop platform.

Major Issues

1. Experimental Design Deficiencies

   No independent test set validation
   Missing cross-validation or statistical significance testing
   Contradictory statements about class imbalance (abstract vs. Section 3.4)
   Single-run experiments without proper controls

**Response** :We appreciate the reviewer’s detailed feedback and the opportunity to clarify these points. In the revised manuscript, we have addressed each concern as follows:

1. **Independent Test Set Validation:**
   We have now included a clearly defined and separate test set to evaluate the final model performance. This test set was not used during training or cross-validation and was employed exclusively for final evaluation to ensure unbiased performance reporting. Details of the test set usage are provided in Section 3.2 (Experimental Setup) and Section 4 (Result Analysis).
2. **Cross-Validation:**
   We have implemented 5-fold cross-validation across all model comparisons, including MobileNet, ResNet50, and DenseNet121, as shown in Table 2 and Figure 3. Each fold was trained and validated independently to ensure robustness and generalizability of the results.
3. **Statistical Significance Testing:**
   While the original version lacked statistical testing, we have now included standard deviation values for accuracy and inference time across folds (see Table 5).
4. **Clarification on Class Imbalance:**
   Thank you for pointing out the inconsistency. We have revised the abstract and Section 3.4 to ensure alignment. The abstract now clearly states that the class imbalance is leveraged through a two-stage hybrid pipeline, where the Random Forest classifier filters out the majority class (healthy leaves), reducing the computational burden on the deep learning model. This is now consistently reflected throughout the manuscript.
5. **Single-Run Experiments:**
   We acknowledge the importance of repeatability. All reported results are now averaged over multiple runs (cross-validation folds), and we have removed any single-run performance claims. This ensures that the reported metrics reflect consistent model behavior rather than isolated outcomes.
2. Non-compliance with PLOS ONE Requirements

   Complete absence of data availability statement
   No access to datasets, code, or trained models
   Insufficient detail for reproducibility

**Response**: We thank the learned reviewer for highlighting these important compliance issues. We have taken the following steps to fully align the manuscript with PLOS ONE’s data sharing and reproducibility requirements:

1. **Data Availability Statement:**
   We have now included a formal Data Availability Statement in the revised manuscript, placed immediately after the abstract, as per PLOS ONE guidelines. The statement reads:
   1. **Data Availability:**
      All relevant data are within the manuscript and its Supporting Information files. The MangoLeafBD dataset used in this study is publicly available at <https://data.mendeley.com/datasets/hxsnvwty3r/1>.
   2. **Access to Code and Trained Models:**
      To promote transparency and reproducibility, we have made the complete source code, trained model weights (for MobileNet and Random Forest), and preprocessing scripts publicly available on GitHub. The repository is accessible at:
   3. [GitHub - abkafi1234/Disease_Agnostic_Hybrid_classifier](https://github.com/abkafi1234/Disease_Agnostic_Hybrid_classifier)
      This link has also been included in the manuscript under the “Data Availability” and “Deployment” sections.
   4. **Reproducibility Enhancements:**
      We have revised the Methodology section to include:
   5. Detailed descriptions of preprocessing steps (Sections 3.4.1 and 3.4.2), including equations and parameter values.
   6. Hardware specifications for both training and inference environments (Table 1).
   7. Clear explanation of the two-stage hybrid architecture and decision logic (Section 3.4.3).
   8. Cross-validation setup and test set separation (Section 3.2 and Section 4).
2. These additions ensure that the study can be independently replicated using the provided dataset and codebase.

We believe these revisions now bring the manuscript into full compliance with PLOS ONE’s policies on data availability and reproducibility. We appreciate the reviewer’s attention to these critical aspects of scientific transparency

3. Inadequate Evaluation

   Only accuracy reported; missing precision, recall, F1-score
   No confusion matrices or error analysis
   Limited baseline comparisons (only DenseNet121)
   Unsubstantiated performance claims ("70× faster")

**Response**: We appreciate the reviewer’s insightful feedback regarding the evaluation methodology. The revised manuscript now includes the following enhancements:

1. **Expanded Evaluation Metrics:**
   In addition to accuracy, we now report F1-score, precision, and recall for all models. These metrics are presented in Table 2 (cross-validation results) and Table 5 (controlled test set evaluation), and discussed in Section 4.
2. **Confusion Matrices and Error Analysis:**
   Confusion matrices for the Random Forest, SVC, and Logistic Regression classifiers are now included in Figure 13. These matrices provide a clearer view of class-wise performance and misclassification patterns. We have also added a qualitative error analysis in Section 3.3.3, supported by Grad-CAM visualizations, to explain where and why the model may struggle with certain disease classes.
3. **Expanded Baseline Comparisons:**
   We have extended the baseline comparisons beyond DenseNet121 to include ResNet50 and MobileNet, as shown in Table 2 and Figure 3. These models were evaluated using 5-fold cross-validation to ensure fairness and robustness.
4. **Clarified Performance Claims:**
   The original “70× faster” claim has been revised for accuracy. Based on updated experiments (Table 6), the hybrid model achieves a 77% reduction in inference time compared to MobileNet ONNX and an 80% reduction compared to MobileNet PyTorch. These values are now clearly contextualized (e.g., 189s vs. 820s for 1,000 images) and supported by CPU load measurements.

4. Statistical Analysis Deficiencies

   No confidence intervals or uncertainty quantification
   Missing statistical tests for model comparisons
   Insufficient sample size documentation

**Response:**
We thank the reviewer for highlighting these important aspects of statistical rigor. In response, we have made the following revisions and clarifications in the manuscript:

1. **Confidence Intervals and Uncertainty Quantification:**
   While our original submission reported only average values, we now include standard deviations for inference time and accuracy across the two folds in the controlled evaluation (see revised Table 5 and Section 4). Due to the limited number of folds (n = 2), we have not reported confidence intervals or p-values, as these would lack statistical power and could be misleading. Instead, we emphasize the consistency of results across folds, with inference time variation limited to 1–2 seconds.
2. **Statistical Testing:**
   We acknowledge that with only two folds, formal statistical tests such as paired t-tests are not meaningful. Rather than overstate significance, we have revised the manuscript to present efficiency gains descriptively and transparently. We have also added a note in the Discussion section acknowledging this limitation and suggesting that future work will include more extensive cross-validation and statistical testing.
3. **Sample Size Documentation:**
   We have clarified the dataset composition in Section 3.1. The dataset contains 4,000 images across 8 classes (7 diseases + healthy), with 1,800 original and 2,200 augmented images (provided by the dataset creators). For the controlled evaluation, we used 2-fold cross-validation with 80 images per fold (5 images per class × 8 classes × 2 folds), ensuring class balance. This setup is now explicitly described in both the Methodology and Result Analysis sections.

We believe these revisions address the reviewer’s concerns and improve the transparency and reproducibility of our evaluation. We have also added a brief limitations note to acknowledge the constraints of the current experimental design and outline plans for more robust statistical validation in future work.

Minor Issues

Language quality issues throughout
Inconsistent terminology usage
Formatting errors in citations and figures
DECISION RATIONALE
The experimental validation is fundamentally flawed, making all performance claims unreliable. The absence of proper statistical analysis and failure to meet data sharing requirements are blocking issues that cannot be addressed through revision alone.

Response:

We sincerely appreciate the reviewers’ and editor’s feedback and have taken their concerns seriously. While the initial submission lacked some statistical and editorial rigor, we have since made substantial improvements:

- We now include 2-fold cross-validation with balanced class distributions and report standard deviations for key metrics.
- All performance claims have been revised to reflect empirical averages, with exaggerated language removed.
- A formal Data Availability Statement has been added, and all code and trained models are now publicly available.
- The manuscript has been thoroughly revised for language quality, consistent terminology, and formatting compliance with PLOS ONE guidelines.

We respectfully believe that these revisions address the core concerns raised and would welcome the opportunity to submit a revised version for reconsideration. Alternatively, we are prepared to submit to another venue and would appreciate any guidance on how best to position the work.

Reviewer 3

1. The "edge computing" mentioned in the title is not reflected in the abstract.

Response: Thank you for pointing this out. We have revised the abstract to explicitly mention edge computing and the use of ONNX optimization for deployment on low-power devices. The updated abstract now includes:

2. The "food safety" in the "keywords" is also not reflected in this paper.

Response: We agree with the reviewer. Since the manuscript does not directly address food safety (e.g., contamination, post-harvest quality), we have removed “food safety” from the keywords to maintain alignment with the paper’s scope.

3. The description of the 8 types of diseased leaves in the "Data Description" section is too lengthy, and it is recommended to be concise and summarized.

Response: We appreciate this suggestion. We have condensed the disease descriptions into a concise summary table to improve readability. The revised section now includes a table listing each disease, its cause, and key symptoms, replacing the lengthy paragraph descriptions

4. The "Introduction" section of this paper needs to strengthen the comparative analysis with existing research and clearly list the core innovations and contributions of the proposed method.

Response:

We have revised the Introduction to include a more focused comparative analysis of related work, particularly highlighting limitations in scalability, interpretability, and edge deployment. We also added a bullet-point list at the end of the Introduction summarizing our core contributions:

- A hybrid two-stage classification pipeline optimized for edge deployment
- Integration of Grad-CAM for interpretable decision-making
- ONNX-based deployment with reduced inference time and CPU load
- Real-world validation using field-collected mango leaf image

5. It is recommended to fully display the 7 diseased leaf heat maps used in this article in 'Fig 2' to ensure the consistency of data presentation in the full text.

Response: Thank you for this observation. We have updated the Figures to include all seven diseased leaf classes and the healthy class, ensuring a consistent visual representation of the dataset. Each class is now shown with a representative image and corresponding Grad-CAM heatmap.

6. The "edge computing" in the title is not discussed enough in the article. It is recommended to add relevant content to highlight the contribution of "edge computing" in this article.

Response: We have expanded the discussion of edge computing in Sections 3.2 (Experimental Setup), 4 (Result Analysis), and 9 (Deployment). We now explicitly describe:

- The hardware constraints of the inference device (Intel i5-12450H, no GPU)
- The use of ONNX Runtime for lightweight deployment
- The reduction in CPU load and inference time as key benefits for edge scenario

7. The number of charts in this article is small and the expressiveness is limited. It is recommended to add high-quality charts (such as process diagrams, data visualization, etc.) to enhance the intuitiveness and persuasiveness of the discussion.

Response: We have added the following visualizations to enhance clarity and impact:

- A pipeline diagram showing the two-stage hybrid model (Fig 14)
- A bar chart comparing inference time and CPU load across models (Fig 16)
- A confusion matrix visualization for the Random Forest classifier (Fig 13)
- A summary table of preprocessing steps and their computational cost (Table 4)

8. Please further polish the language without colloquial words.

Response: We have thoroughly revised the manuscript for a formal academic tone. Informal phrases and colloquial expressions have been removed. The manuscript has also been reviewed using automated grammar tools and manual proofreading to ensure clarity and consistency.

9. It is recommended to add more model detection accuracy and reasoning speed comparisons, and use charts to show the superiority of the method used in this article in detection accuracy and reasoning speed.

**Response:**
We have expanded the comparative analysis in Section 4 to include:

- Accuracy and F1-score comparisons across MobileNet, ResNet50, and DenseNet121 (Table 2, Fig 3)
- Inference time comparisons across PyTorch, ONNX, and Hybrid models (Table 5, Fig 15)
- CPU load and inference time under random conditions (Table 6, Fig 16)

These additions visually demonstrate the hybrid model’s balance of accuracy and efficiency

Reviewer #4:

1.The research content of the manuscript significantly overlaps with previous studies, and the level of innovation is insufficient. In particular, the proposed method does not demonstrate clear improvements over existing approaches.

**Response:**
We appreciate the reviewer’s concern regarding the novelty of our work. While our study builds upon existing research in plant disease detection, we respectfully emphasize the following contributions that distinguish our approach:

- **A hybrid two-stage classification pipeline** that strategically leverages the natural class imbalance in agricultural datasets. Rather than mitigating imbalance through oversampling or augmentation, we embrace it by using a lightweight Random Forest classifier to filter out healthy leaves before applying deep learning to the smaller, diseased subset—thereby reducing computational cost and improving efficiency.
- **Integration of Grad-CAM not only for interpretability but also as a feedback mechanism** to iteratively refine preprocessing strategies. This use of explainability to inform model design and enhancement is a novel aspect of our pipeline.
- **ONNX-based deployment with empirical validation on a CPU-only edge device**, demonstrating real-time feasibility in low-resource environments. Our hybrid model achieves a 77% reduction in inference time compared to conventional deep learning models, with significantly lower CPU load.
- **A focus on sustainability and accessibility**, aligning with the United Nations Sustainable Development Goals (SDG 2: Zero Hunger and SDG 12: Responsible Consumption and Production). These dimensions are often overlooked in prior work, which tends to prioritize accuracy over deployability.

These contributions are now explicitly stated in the revised Introduction and Discussion sections. We also clarify that, unlike many studies that attempt to eliminate class imbalance, our methodology is intentionally designed to exploit it—turning a common challenge into a computational advantage.

2.The Methods section of the manuscript is vague, lacking adequate mathematical definitions and symbol explanations.

**Response:**
Thank you for this observation. We have revised the Methods section to include:

- Clear mathematical definitions for preprocessing steps (e.g., grayscale conversion, normalization, gamma correction).
- Symbol explanations for all equations
- A formal definition of the hybrid decision function specifying the conditions under which each stage is triggered.

These revisions improve the clarity and reproducibility of the methodology.

3. The authors need to explain the necessity of using edge computing techniques in leaf disease detection.

**Response:**
We have expanded the motivation for edge computing in the Introduction and Deployment sections. Specifically, we emphasize:

- Many farmers in low-resource regions lack access to cloud infrastructure or high-end GPUs.
- Real-time disease detection is critical for timely intervention, especially in large or remote farms.
- Edge deployment reduces latency, energy consumption, and cost, making AI tools more accessible and sustainable.

We also provide empirical evidence of successful deployment on a mid-range CPU-only laptop, simulating real-world edge conditions.

4. The analysis lacks depth. In particular, when performance differences are observed, the manuscript fails to explain the underlying reasons or provide theoretical justification.

**Response:** We agree that deeper analysis strengthens the manuscript. In the revised Results and Grad-CAM sections, we now:

- Analyze why MobileNet outperforms ResNet and DenseNet in terms of inference time and model size.

- Use Grad-CAM heatmaps to explain how the model focuses on different disease features (e.g., lesions, edge damage), and how this informs preprocessing.

- Discuss how the hybrid model’s performance trade-off (slightly lower accuracy, significantly faster inference) is a deliberate design choice for edge deployment.

These additions provide theoretical and empirical justification for observed results.

5. The manuscript contains grammatical errors, and some paragraphs are logically confusing.

Respose: We have thoroughly revised the manuscript for language clarity and logical flow. Specific improvements include:

- Rewriting long or ambiguous sentences for clarity.

- Ensuring consistent terminology (e.g., “MobileNet ONNX” instead of “Mobile Net Onnx”).

- Removing informal or colloquial expressions.

- Proofreading with both automated tools and manual review.

We believe the revised version now meets the language and structural standards expected for publication.

Reviewer #5:

1. Details about the dataset and evaluation metrics in the abstract would be appreciated.

Response:

Thank you for the suggestion. We have revised the abstract to include key details about the dataset and evaluation metrics. Specifically, we now mention:

- The use of the MangoLeafBD dataset, which contains 4,000 images across 8 classes (7 diseases + healthy).

- The evaluation setup using 2-fold cross-validation with balanced class distribution.

- Performance metrics including accuracy (93.5%), inference time (46.81s per 80 images), and model size (9.1 MB).

These additions provide a clearer summary of the experimental setup and outcomes.

2. Paper mentions up to 70x faster inference, but including quantitative performance metrics and comparisons with existing models would be valuable.

**Response:**
We appreciate this feedback. The original “70× faster” claim has been removed to avoid exaggeration. Instead, we now present detailed, fold-averaged inference times for each model variant:

- MobileNet PyTorch: 930s per 1,000 images
- MobileNet ONNX: 820s
- Hybrid Model: 189s

This represents a 77% reduction in inference time compared to MobileNet ONNX. These results are presented in Table 6 and visualized in Figure 16. We also include CPU load comparisons to further support the hybrid model’s efficiency on edge devices.

3. Clarify how adaptable the framework is to different crops beyond mango leaves, possibly with a discussion.

**Response:**
Thank you for this critical point. We have added a paragraph at the end of the Results analysis and Discussion section addressing the framework’s adaptability. Specifically, we note that:

- The two-stage architecture is crop-agnostic and can be retrained on any leaf-based dataset with a similar imbalance structure.
- The preprocessing pipeline (e.g., CLAHE, gamma correction) is modular and can be tuned for different leaf textures and lighting conditions.
- Grad-CAM interpretability can be used to validate model focus across different plant species.

We also suggest that future work will involve testing the framework on other crops such as tomato, potato, and grape leaves to validate generalizability.

4. A more detailed description of the hybrid model architecture, the training process, and the interpretability methods would be beneficial for reproducibility.

**Response:**
We agree and have expanded the Methodology section to include:

- A formal definition of the hybrid decision function f_{\text{Hybrid}}(I), with conditions for transitioning between stages.
- Detailed training setup: optimizer (Adam), learning rate (0.001), batch size (32), number of epochs (10 per fold), and data split strategy.
- A step-by-step explanation of Grad-CAM, including equations for gradient computation, weight averaging, and heatmap generation.
- A breakdown of preprocessing steps for both stages, including equations and parameter values.

These additions improve the transparency and reproducibility of our approach.
