# Supplementary material for: LeafAI: Interpretable plant disease detection for edge computing
Source: PLoS One. 2026 Jan 23;21(1):e0335956. doi: 10.1371/journal.pone.0335956 (PMC12829967; doi:10.1371/journal.pone.0335956)
Supplement: Attachment — Submitted filename: Response to the reviewer.docx [file pone.0335956.s003.docx]

1. The experimental validation lacks sufficient statistical rigor, as the authors rely on only 2-fold cross-validation for the controlled evaluation and 5-fold for initial comparisons, without reporting confidence intervals, p-values, or more robust tests like paired t-tests to confirm the significance of performance differences between models. This limits the reliability of claims such as the 77% inference time reduction, especially given the small sample sizes (e.g., 80 images per fold), and could be addressed by expanding to 10-fold validation or bootstrapping methods for better uncertainty quantification.

   **Answer:** We appreciate the reviewer’s concern regarding statistical rigor. In the revised experiments, we have expanded our evaluation to 10-fold cross-validation and increased the dataset to a total of 600 images, which provides a more robust assessment of model performance. Additionally, we now report confidence intervals for key metrics to quantify uncertainty and better support claims, such as the 77% reduction in inference time. These changes enhance the reliability and statistical validity of our results, addressing the concerns about small sample sizes and limited cross-validation.
2. The comparison with baseline models is limited to only three architectures, without including state-of-the-art alternatives like EfficientNet or YOLO-based detectors tailored for agricultural tasks, which may overlook potential superior performers in efficiency or accuracy. Expanding baselines would strengthen the paper's claims of superiority and provide a more comprehensive evaluation.

**Answer:** We thank the reviewer for this valuable suggestion. In the revised manuscript, we have expanded the set of baseline models to include EfficientNet, a strong state-of-the-art architecture for image classification tasks, which provides a meaningful comparison in terms of both accuracy and efficiency. While object detection frameworks such as YOLO are highly impactful in agricultural applications, our study focuses specifically on the classification setting. For this reason, we prioritized including architectures that are directly optimized for classification. We believe the addition of EfficientNet strengthens the comprehensiveness of our evaluation and further supports the claims of our proposed method’s effectiveness.

3. The preprocessing rationale, while informed by Grad-CAM, does not sufficiently justify the choice of specific techniques through ablation studies or sensitivity analysis, leaving readers unclear on how parameter variations impact performance. Conducting ablation experiments to isolate the contribution of each preprocessing step would enhance the methodological transparency and reproducibility.

**Answer:** We appreciate the reviewer’s suggestion regarding ablation studies for preprocessing. To address this, we conducted a detailed analysis to understand the performance improvements of the hybrid model. A **random sample** from the test set was used to examine the model’s behavior and the impact of preprocessing. The results, now included in the manuscript, demonstrate that the chosen preprocessing steps effectively preserve relevant features, thereby supporting the hybrid model’s efficiency and accuracy. This study demonstrates the role of preprocessing while maintaining the focus on the improvements of the hybrid model.

4. The paper claims adaptability to other crops but provides no empirical evidence, such as transfer learning results on datasets like PlantVillage for tomatoes or potatoes, relying instead on qualitative assertions. To substantiate generalizability, the authors should include cross-dataset experiments or fine-tuning results to demonstrate the framework's robustness beyond mango leaves.

**Answer:** We thank the reviewer for highlighting the importance of demonstrating generalizability. While the current study focuses on mango leaves, which are a key crop in the Indian subcontinent, the proposed pipeline is designed with adaptability in mind. Empirical testing on other crops with similar disease profiles is a valuable next step and is planned for future work. Additionally, cross-dataset experiments or fine-tuning on datasets such as PlantVillage could further validate robustness, but were beyond the scope of the present study. We have clarified these points in the manuscript to set realistic expectations and outline directions for extending the framework to other crops and larger datasets.

5. Review: While the deployment on a laptop simulates edge computing, it uses a relatively powerful Intel i5-12450H processor, which does not fully reflect ultra-low-power devices like Raspberry Pi or NVIDIA Jetson Nano, common in true field conditions. Testing on such hardware, including metrics like energy consumption and real-time latency with live camera feeds, would better validate practical applicability in remote agricultural settings.

**Answer:** We emphasize that our study does not focus on ultra–low-power devices, nor do we make such claims. From the introduction and consistently throughout, we clearly position our work on commodity hardware, particularly laptops, because they are widely available, affordable, and versatile. This choice aligns with our goal of supporting farmers and advancing the United Nations’ Sustainable Development Goals (SDG 2: Zero Hunger and SDG 12: Responsible Consumption and Production). A single laptop can handle diverse workloads for a farmer, whereas requiring them to purchase a Raspberry Pi (≈ USD 100 for a complete kit) or a Jetson Nano (≈ USD 200) would undermine the very premise of using commodity hardware. Moreover, neither Raspberry Pi nor Jetson Nano can be considered commodity hardware in the same sense as laptops.

A growing body of research further supports this design choice. Early work has demonstrated that the Raspberry Pi struggles with deep learning inference due to CPU-only limitations, latency, and memory bottlenecks [1]. Subsequent benchmarking studies reinforced this finding, showing that the Raspberry Pi significantly underperforms in terms of throughput and energy efficiency compared to accelerator-equipped edge devices [2]. Later evaluations highlighted that Pi scores poorly on energy–latency trade-offs, further emphasizing its inefficiency for real-world ML inference[3].

Even attempts to adapt deep learning to Raspberry Pi reveal its unsuitability. Lightweight CNNs and pruning/quantization techniques are often required just to achieve acceptable performance, but this comes at the expense of accuracy and general applicability [4].More recent empirical studies show that running heavy CNNs such as ResNet or DenseNet on Raspberry Pi 4 results in excessively high inference times and memory limitations, making only very small models feasible [5]. Additional research highlights further software and runtime limitations, including incomplete support for quantized operators, which complicates deployment [6]

Comprehensive reviews also confirm these observations: Raspberry Pi is valuable for prototyping but suffers from thermal throttling, power inefficiency, and compute constraints when applied to production-grade AI [7]. Broader surveys on sustainable AI similarly note that the energy and hardware constraints of devices like Raspberry Pi severely restrict the scope of deployable models and scenarios [8]. Finally, head-to-head comparisons with Jetson boards and Coral accelerators consistently show that Pi cannot sustain throughput under real workloads due to thermal throttling and CPU-only execution. [8].

In contrast, laptops are genuine commodity hardware. Even with older components, they deliver practical performance. For example, the Intel Core i5-12450H we use is already three years old, yet it demonstrates feasibility. To address lower-power scenarios, we further benchmarked a laptop equipped with an 8th-generation Intel i5 processor operating at a modest 15-watt TDP, showing that commodity laptops remain a far more realistic and sustainable platform for our target use case than Raspberry Pi or Jetson Nano.

6. The integration of explainable AI via Grad-CAM is promising. Still, the analysis remains qualitative, lacking quantitative metrics such as Intersection over Union (IoU) between heatmaps and ground-truth disease annotations to measure explanation fidelity. Incorporating such metrics would provide objective evidence of interpretability improvements and their role in iterative refinement.

**Answer:** We thank the reviewer for recognizing the potential of Grad-CAM in our study. In our work, Grad-CAM is employed layer-wise to identify the model’s focus and guide the selection of preprocessing techniques, rather than as a quantitative evaluation tool. While we do not report metrics such as IoU with ground-truth disease annotations, the qualitative analysis provides actionable insights that directly informed the design of the hybrid model. We have clarified this rationale in the manuscript to emphasize the purpose of Grad-CAM in supporting preprocessing and model interpretability.

7. The paper overlooks potential biases in the MangoLeafBD dataset, such as geographic specificity to Bangladesh or augmentation artifacts, which could affect model generalization; a deeper discussion of bias mitigation strategies, like fairness audits or diverse data sourcing, is needed to ensure equitable performance across global agricultural contexts. Furthermore, to address similar challenges in defect detection and class imbalance, the authors are recommended to cite and compare with "Enhancing grid reliability through advanced insulator defect identification" (https://doi.org/10.1371/journal.pone.0307684) and "Deep Learning-Based Integrated Circuit Surface Defect Detection: Addressing Information Density Imbalance for Industrial Application" (https://doi.org/10.1007/s44196-024-00423-w), which offer insights into handling imbalanced data and defect localization in industrial applications that parallel agricultural disease detection.
8. **Answer:** We appreciate the reviewer’s insightful comments regarding potential dataset biases and generalization. We have now added the recommended citations to contextualize strategies for handling class imbalance and defect localization, which are relevant to agricultural disease detection.

While testing on datasets from other countries could further validate generalizability, mango production is primarily concentrated in the Indian subcontinent, making the current MangoLeafBD dataset a strong foundation for evaluation. We have clarified this in the manuscript. Additionally, the current implementation processes images sequentially, which limits throughput for large datasets or real-time applications. Future work will explore parallel processing strategies to enhance scalability and efficiency under higher data loads.

References.

1.	Velasco-Montero D, Fernández-Berni J, Carmona-Galán R, Rodríguez-Vázquez A. Performance Analysis of Real-Time DNN Inference on Raspberry Pi.

2.	Alvarado Rodriguez O, Dave D, Liu W, Su B. A Study of Machine Learning Inference Benchmarks. 2020 [cited 26 Sep 2025]. doi:10.1145/3441250.3441277

3.	Tu X, Mallik A, Chen D, Han K, Altintas O, Wang H, et al. Unveiling Energy Efficiency in Deep Learning: Measurement, Prediction, and Scoring Across Edge Devices. Proceedings - 2023 IEEE/ACM Symposium on Edge Computing, SEC 2023. 2023;1: 80–93. doi:10.1145/3583740.3628442

4.	Ameen S, Siriwardana K, Theodoridis T. Optimizing Deep Learning Models For Raspberry Pi.

5.	Tank MB, Patel M, Deshmukh K. Evaluating Deep Learning Model Performance on Raspberry Pi 4 for COVID-19 Diagnosis. Journal of Information Systems Engineering and Management. 2025;10: 722–736. doi:10.52783/JISEM.V10I29S.4587

6.	Balemans D, Vandersmissen B, Steckel J, Mercelis S, Reiter P, Oramas J. Deep Learning Model Compression for Resource Efficient Activity Recognition on Edge Devices: A Case Study. 2024. doi:10.5220/0012423300003660

7.	Mathe SE, Kondaveeti HK, Vappangi S, Vanambathina SD, Kumaravelu NK. A comprehensive review on applications of Raspberry Pi. Comput Sci Rev. 2024;52: 100636. doi:10.1016/J.COSREV.2024.100636

8.	Minott D, Siddiqui S, Haddad RJ. Benchmarking Edge AI Platforms: Performance Analysis of NVIDIA Jetson and Raspberry Pi 5 with Coral TPU. Conference Proceedings - IEEE SOUTHEASTCON. 2025; 1384–1389. doi:10.1109/SOUTHEASTCON56624.2025.10971592
